# Microvascular Outcomes of Glucagon-Like Peptide-1 (GLP-1) Receptor Agonists in Type 2 Diabetes: A Systematic Review of Retinopathy and Nephropathy Evidence

**DOI:** 10.7759/cureus.92976

**Published:** 2025-09-22

**Authors:** Atia Arif, Sanu Lama, Bhavna Singla, Shivam Singla, Sunita Kumawat, Anusha Tharwani, Muhammad Usman, Hamna Khalid, Venkata Madusudana Rao Kanukollu, Osatohanmwen Ekomwereren, Shabir Khan

**Affiliations:** 1 Psychiatry and Behavioral Sciences, Sialkot Medical College, Sialkot, PAK; 2 Research, Prime Healthcare, Ontario, USA; 3 Internal Medicine, Janaki Medical College and Teaching Hospital, Janakpur, NPL; 4 Internal Medicine, Erie County Medical Center Health Campus, Buffalo, USA; 5 Internal Medicine, TidalHealth Penninsula Regional, Salisbury, USA; 6 Internal Medicine, Hackensack Meridian Ocean Medical Center, New Jersey, USA; 7 Internal Medicine, Jinnah Postgraduate Medical Center, Karachi, PAK; 8 Internal Medicine, King Edward Medical University, Lahore, PAK; 9 Ophthalmology, Stoke Mandeville Hospital, Aylesbury, GBR; 10 Trauma and Orthopaedics, Shrewsbury and Telford Hospital NHS Trust, Shrewsbury, GBR; 11 Internal Medicine, CMH Lahore Medical and Dental College, Lahore, PAK

**Keywords:** diabetic nephropathy, diabetic retinopathy, glp-1 receptor agonists, microvascular complications, randomized controlled trials, type 2 diabetes

## Abstract

This systematic review evaluated randomized controlled trials examining the effects of glucagon-like peptide-1 (GLP-1) receptor agonists on microvascular outcomes in type 2 diabetes, focusing on diabetic retinopathy and nephropathy. Four eligible RCTs, enrolling over 27,000 patients with follow-up periods ranging from 32 weeks to 5.4 years, were included. GLP-1 receptor agonists consistently demonstrated renal protective effects, primarily driven by reductions in new or worsening nephropathy and macroalbuminuria, with more modest and inconsistent effects on estimated glomerular filtration rate (eGFR) decline. In contrast, their impact on retinopathy remained inconclusive. A transient signal of worsening retinopathy has been reported in the context of rapid glycemic improvement; however, across large outcome trials, effects on retinopathy have been inconsistent and remain inconclusive. Overall, the evidence for retinopathy risk is limited by small event numbers, heterogeneity in assessments, and exploratory analyses. The certainty of renal benefit was strengthened by rigorous trial designs and low risk of bias, whereas retinopathy outcomes were generally secondary and less robust. These findings suggest that GLP-1 receptor agonists can be prioritized for patients at high renal risk, but careful monitoring of individuals with pre-existing retinopathy remains warranted.

## Introduction and background

Type 2 diabetes mellitus (T2DM) is a global health challenge, affecting over 500 million people worldwide and projected to increase further in the coming decades [1]. Among its most disabling and costly consequences are microvascular complications, primarily diabetic retinopathy (DR) and diabetic nephropathy (DN). Diabetic retinopathy remains the leading cause of preventable blindness in working-age adults [2], while diabetic nephropathy is the leading cause of chronic kidney disease (CKD) and end-stage renal disease (ESRD) worldwide [3]. These complications substantially reduce quality of life and increase cardiovascular morbidity and mortality.

While intensive glycemic control has been shown to reduce the risk of microvascular complications, the extent of benefit and the balance with treatment-related risks may vary depending on the glucose-lowering agent used [4,5]. Beyond lowering glucose, modern antihyperglycemic therapies, particularly glucagon-like peptide-1 (GLP-1) receptor agonists (GLP-1 RAs), have demonstrated cardiovascular safety and efficacy, as well as favorable effects on weight and blood pressure. However, their impact on microvascular outcomes, especially retinopathy and nephropathy, is less well defined [6,7]. Importantly, although some large cardiovascular outcome trials have reported microvascular endpoints, these were typically secondary analyses, and to date, no randomized controlled trial has been specifically powered or primarily designed to assess diabetic retinopathy progression. This limitation contributes to the ongoing uncertainty in the evidence base and underscores the need for a systematic evaluation.

Given this emerging but inconsistent body of evidence, it is crucial to systematically examine the available randomized controlled trials (RCTs) to better understand the risks and benefits of GLP-1 RAs with respect to microvascular complications. This systematic review aims to evaluate the effects of GLP-1 RAs compared with standard care on the risks of diabetic retinopathy and diabetic nephropathy in patients with type 2 diabetes, focusing on evidence from randomized clinical trials.

## Review

Materials and methods

Study Design and Protocol Registration

This systematic review was conducted in accordance with the Preferred Reporting Items for Systematic Reviews and Meta-Analyses (PRISMA) guidelines [8]. A predefined protocol was developed to ensure transparency and methodological rigor. The review focused on randomized controlled trials (RCTs) assessing the effects of GLP-1 receptor agonists on microvascular outcomes, specifically diabetic retinopathy and nephropathy.

Eligibility Criteria (PICO - Patient, Intervention, Comparison, Outcome - Framework)

The study question was framed using the PICO approach [9]. The population included adults with type 2 diabetes mellitus (T2DM), irrespective of baseline cardiovascular or microvascular disease status. The intervention was treatment with a GLP-1 receptor agonist at any approved dose or regimen. The comparator was placebo or standard care, with or without background therapy. The outcomes of interest were diabetic retinopathy events (progression, complications, or surrogate retinal measures) and nephropathy outcomes (incident or worsening albuminuria, eGFR decline, renal composite events, or surrogate kidney markers). Only randomized controlled trials reporting original data were eligible to ensure the highest level of evidence and minimize confounding. Observational studies, narrative reviews, and non-randomized designs were excluded. However, given the limited number of eligible RCTs, the potential role of high-quality observational data should be acknowledged. Such studies, particularly large registries or well-designed cohorts, may provide valuable complementary evidence for rarer outcomes like retinopathy progression and could be considered in future reviews to broaden the understanding of real-world risks and benefits.

Information Sources and Search Strategy

A comprehensive literature search was undertaken in PubMed/MEDLINE, Embase, and the Cochrane Central Register of Controlled Trials (CENTRAL), from inception until the most recent update in 2025. The search combined controlled vocabulary (e.g., MeSH terms) and free-text keywords relating to “GLP-1 receptor agonist,” “diabetic retinopathy,” and “diabetic nephropathy.” Reference lists of relevant reviews and included studies were hand-searched to identify additional eligible trials. No language restrictions were applied.

Study Selection

All identified records were imported into a reference manager, and duplicates were removed. Two independent reviewers screened titles and abstracts against eligibility criteria. Full-text articles were then retrieved for detailed assessment, with disagreements resolved by consensus or by consultation with a third reviewer. The PRISMA flow diagram was used to document the selection process, including reasons for study exclusion.

Data Extraction and Management

A standardized extraction form was used to capture key study characteristics. Extracted data included trial design, sample size, baseline population characteristics, intervention details (drug, dose, duration), comparator, definitions of retinopathy and nephropathy outcomes, follow-up duration, and reported effect estimates (hazard ratios, risk ratios, mean differences with confidence intervals). When necessary, supplementary material was consulted to obtain complete information.

Risk of Bias Assessment

The Cochrane Risk of Bias 2.0 tool [10] was applied to assess methodological quality of included RCTs. Domains evaluated included randomization process, deviations from intended interventions, missing outcome data, measurement of outcomes, and selective reporting. Each domain was judged as low risk, some concerns, or high risk. Risk of bias assessments were performed independently by two reviewers, with disagreements resolved through discussion.

Data Synthesis

Given the limited number of eligible studies and heterogeneity in outcome definitions and reporting, a narrative synthesis was chosen over meta-analysis. Studies were grouped according to microvascular outcome type (retinopathy vs. nephropathy) and study design (cardiovascular outcome trials vs. mechanistic trials). Results were critically compared to highlight consistencies, discrepancies, and potential mechanisms. Risk of bias assessments were integrated into the interpretation of findings to ensure balanced conclusions.

Results

Study Selection Process

**Figure 1:**
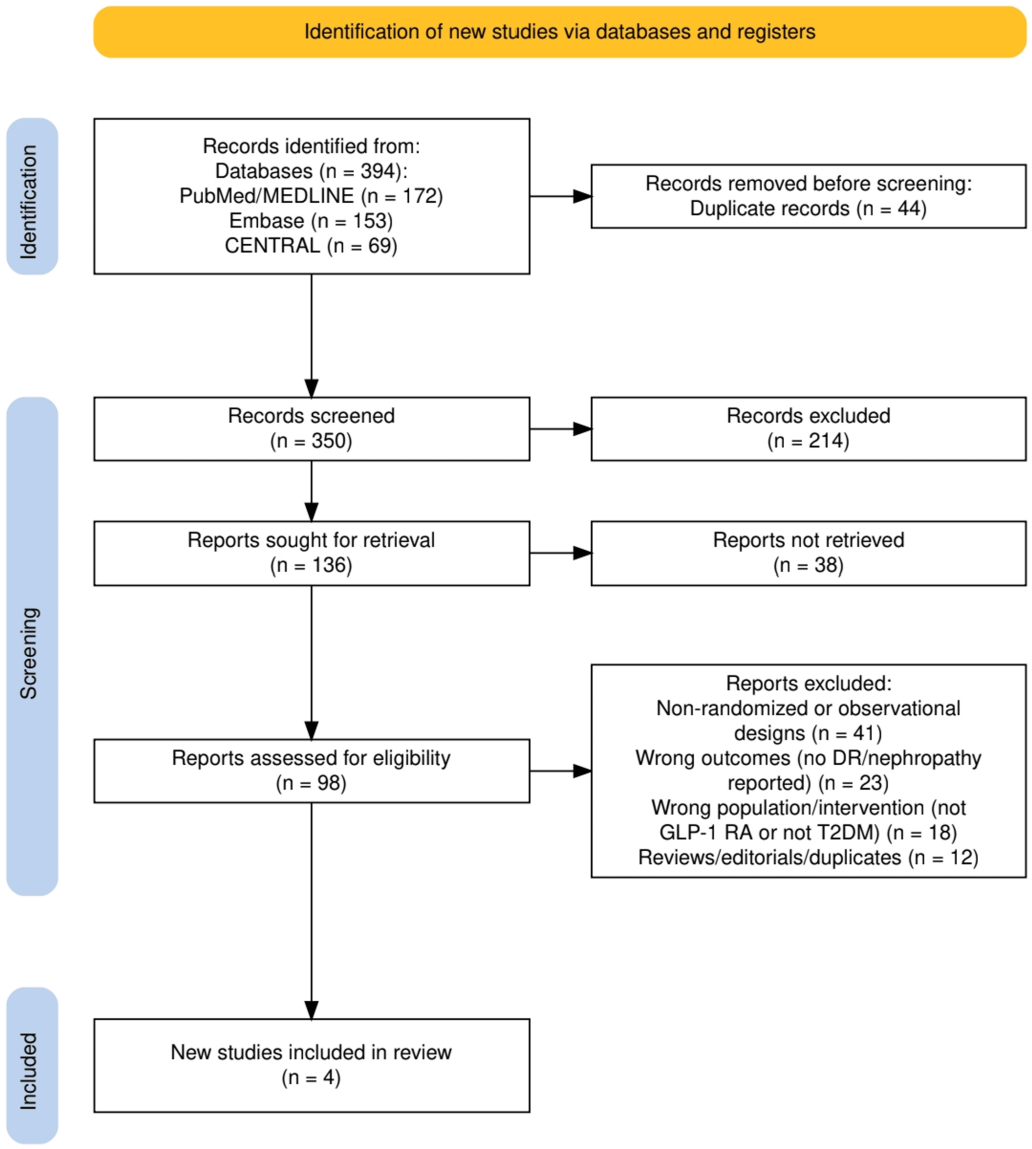
The PRISMA flow diagram is illustrating the process of selection the studies. PRISMA: Preferred Reporting Items for Systematic Reviews and Meta-Analyses

Figure 1 illustrates the PRISMA flow diagram for study selection in this review. A total of 394 records were identified across three databases, including 172 from PubMed/MEDLINE, 153 from Embase, and 69 from CENTRAL. After the removal of 44 duplicates, 350 records underwent screening, of which 214 were excluded at the title and abstract stage. Of the 136 full-text reports sought for retrieval, 38 could not be accessed due to restrictions such as paywalls, inaccessible conference abstracts, or incomplete trial registry records without published results. This left 98 articles for detailed eligibility assessment. Following evaluation, 94 studies were excluded for reasons including non-randomized design (n = 41), absence of relevant outcomes such as diabetic retinopathy or nephropathy (n = 23), wrong population or intervention (n = 18), and reviews or duplicates (n = 12). Ultimately, four randomized controlled trials met the inclusion criteria and were incorporated into the qualitative synthesis.

Characteristics of the Selected Studies

Table 1 summarizes the key characteristics of the four randomized trials included in this review. Collectively, these studies span a range of designs, populations, and follow-up durations, from large cardiovascular outcome trials extending over several years to short-term mechanistic investigations. The populations enrolled varied from broad cohorts of patients with type 2 diabetes and diverse cardiovascular and microvascular risk profiles to smaller, tightly controlled experimental groups. Interventions consistently involved GLP-1 receptor agonists administered at standard therapeutic doses, compared against placebo or active comparators. While one trial demonstrated a signal for worsening diabetic retinopathy complications, others showed either neutral effects or only subtle structural retinal changes of uncertain clinical significance. In contrast, renal outcomes were more consistent across trials, with signals for reduced risk of nephropathy, particularly in larger outcome studies. Overall, the heterogeneity in study design, endpoints, and follow-up highlights both the robustness of renal protection and the ongoing uncertainty surrounding retinopathy outcomes.

**Table 1:** Summary of randomized controlled trials evaluating GLP-1 receptor agonists in diabetic retinopathy and nephropathy. GLP-1 RA: Glucagon-like peptide-1 receptor agonist; RCT: Randomized controlled trial; CVOT: Cardiovascular outcome trial; T2DM: Type 2 diabetes mellitus; CV: Cardiovascular; DR: Diabetic retinopathy; HR: Hazard ratio; CI: Confidence interval; HbA1c: Hemoglobin A1c; QW: Once weekly; eGFR: Estimated glomerular filtration rate; RRT: Renal replacement therapy; EQW: Exenatide once weekly; LSMD: Least squares mean difference; BMI: Body mass index

Study (Author et al., Year)	Design / Duration	Population (n, baseline characteristics)	Intervention (GLP-1 RA, dose)	Comparator	Retinopathy Outcomes	Nephropathy Outcomes	Follow-up	Key Findings
Marso et al., 2016 (SUSTAIN-6) [11]	RCT, CVOT, 2 years	3,297 patients with T2DM, high CV risk; ~29% with baseline DR	Semaglutide 0.5–1.0 mg weekly	Placebo + standard care	↑ DR complications (HR 1.76; 95% CI 1.11–2.78)	↓ New/worsening nephropathy (HR 0.64; 95% CI 0.46–0.88)	Median 2.1 years	Demonstrated CV safety; renal protection; retinopathy worsening signal likely linked to rapid HbA1c reduction
Bethel et al., 2020 (EXSCEL) [12]	RCT, CVOT	13,884 patients with T2DM; broad CV risk spectrum	Exenatide QW 2 mg	Placebo + standard care	No difference between groups; retinopathy rates neutral, unaffected by HbA1c reduction or prior DR status	Macroalbuminuria: HR 0.87 (95% CI 0.70–1.07); Renal composite 1 (≥40% eGFR decline, RRT, or renal death): no significant reduction; Renal composite 2 (includes macroalbuminuria): HR 0.85 (95% CI 0.74–0.98) after adjustment	Median 3.2 years	EQW did not significantly alter eGFR decline; modest renal benefit (macroalbuminuria, adjusted renal composite 2); no effect on DR progression
Kwan et al., 2022 (REWIND, post hoc) [13]	Post hoc analysis of RCT (REWIND), up to 72 months	9,901 patients with T2DM; broad CV and microvascular risk spectrum	Dulaglutide 1.5 mg weekly	Placebo + standard care	Not specifically powered for new/worsening DR; analysis stratified by presence of prior retinopathy. HbA1c reduction consistent irrespective of DR status	Prior nephropathy did not modify HbA1c effect; no direct renal event analysis in this post hoc, but REWIND overall prespecified renal composite (not here)	Median 5.4 years	Dulaglutide reduced HbA1c significantly (LSMD -0.61%) across all strata (duration, DR/nephropathy, BMI). Sustained glycemic benefit. Confirms efficacy regardless of microvascular disease history
Gullaksen et al., 2023 [14]	Prespecified secondary analysis of RCT; 32 weeks	120 adults with T2DM, ≥50 years, established/high CV risk; randomized 30 per group	Semaglutide, Empagliflozin, Combination, or Placebo	Placebo / active comparator arms	No effect on retinal oxygenation, vascular autoregulation, or vessel diameter. Semaglutide increased central retinal thickness slightly (+3.8 μm; ~1%; p=0.009). Clinical significance uncertain.	None assessed (trial focused on retinal and vascular physiology, not renal outcomes).	32 weeks	Exploratory mechanistic evidence: semaglutide may affect retinal thickness but not functional retinal oxygenation/autoregulation. Not powered for clinical DR events.

Quality Assessment

Table 2 presents the quality assessment of the included trials using the RoB 2 framework. Overall, the larger cardiovascular outcome trials demonstrated low risk of bias across key domains, particularly in randomization, blinding, and handling of missing data, which strengthens the reliability of their renal outcome findings. However, the assessment highlighted some concerns regarding retinopathy outcomes, largely because these were not primary endpoints but rather adverse event reports or exploratory analyses, raising the possibility of selective emphasis. This design choice likely reflects practical considerations: retinopathy events were relatively infrequent over the trial durations, and systematic ophthalmologic imaging was not mandated, making robust statistical powering or standardized assessments challenging within cardiovascular-focused trial frameworks. The post hoc nature of one analysis introduced additional uncertainty, as the outcomes were not prespecified in the original statistical analysis plan. In contrast, the smaller mechanistic trial was well-conducted with objective measurements and low attrition, but its limited sample size and reliance on secondary endpoints restricted its ability to inform clinical outcomes. Taken together, the risk of bias is generally low for renal outcomes but more uncertain for retinopathy, which must be considered when interpreting the overall evidence base.

**Table 2:** Risk of bias assessment of included randomized controlled trials. RoB 2: Cochrane Risk of Bias 2 tool; SAP: Statistical analysis plan; BMI: Body mass index; CI: Confidence interval; CV: Cardiovascular; CVOT: Cardiovascular outcome trial; DN: Diabetic nephropathy; DR: Diabetic retinopathy; eGFR: Estimated glomerular filtration rate; EQW: Exenatide once weekly; GLP-1 RA: Glucagon-like peptide-1 receptor agonist; HbA1c: Hemoglobin A1c; HR: Hazard ratio; LSMD: Least squares mean difference; QW: Once weekly; RCT: Randomized controlled trial; RRT: Renal replacement therapy; T2DM: Type 2 diabetes mellitus

Study (Author et al., Year)	Tool Applied	Randomization Process	Deviations from Intended Interventions	Missing Outcome Data	Measurement of Outcomes	Selective Reporting	Overall Risk of Bias
Marso et al., 2016 (SUSTAIN-6) [11]	RoB 2	Low risk – robust RCT randomization in CVOT	Low risk – double-blind, placebo-controlled	Low risk – attrition minimal, balanced	Some concern – retinopathy complications were adverse event reports, not prespecified	Some concern – DR outcomes not primary; exploratory	Some concerns (mainly for DR outcomes; renal outcomes more robust)
Bethel et al., 2020 (EXSCEL) [12]	RoB 2	Low risk – large CVOT with proper randomization	Low risk – double-blind, placebo-controlled	Low risk – excellent follow-up	Low risk – microvascular outcomes prespecified	Low risk – outcomes reported as planned	Low risk
Kwan et al., 2022 (REWIND, post hoc) [13]	RoB 2 (adapted for post hoc)	Low risk – underlying RCT randomization	Low risk – blinded, placebo-controlled	Low risk – attrition small relative to sample size	Low risk – HbA1c and DR/nephropathy history objectively defined	Some concern – post hoc, exploratory analyses not in original SAP	Some concerns (post hoc nature)
Gullaksen et al., 2023 [14]	RoB 2	Low risk – randomization described, small sample	Low risk – partly open-label but outcomes objective (imaging)	Low risk – minimal loss given short follow-up	Low risk – retinal oxygenation/thickness objectively measured	Some concern – secondary outcomes, not powered for clinical DR	Some concerns (secondary endpoints, small size)

Discussion

Across the four included studies, GLP-1 receptor agonists consistently demonstrated renoprotective effects, while their impact on diabetic retinopathy (DR) was more variable. The SUSTAIN-6 trial (Marso et al. [11]) uniquely identified a significant increase in DR complications with semaglutide, in contrast to EXSCEL (Bethel et al., [12]) and REWIND (Kwan et al., [13]), which showed neutral effects on retinopathy outcomes. In SUSTAIN-6, the mean HbA1c reduction with semaglutide was approximately 1.0-1.5% within the first 16 weeks, representing a relatively rapid decline compared with the placebo group. This degree and speed of glycemic improvement have been linked to the phenomenon of “early worsening” of DR, particularly in patients with pre-existing retinopathy.

At the same time, both SUSTAIN-6 [11] and EXSCEL [12] reported meaningful reductions in nephropathy, especially macroalbuminuria and composite renal outcomes, while REWIND [13] confirmed durable glycemic control across patient subgroups regardless of prior microvascular disease. Gullaksen et al. [14] added mechanistic insights, showing small changes in retinal thickness with semaglutide but no functional impairment. Together, these findings support a strong signal for renal protection with GLP-1 RAs, whereas the question of retinopathy risk remains unresolved and context-dependent.

The evidence base is limited by substantial heterogeneity in study design and endpoints. SUSTAIN-6 [11] and EXSCEL [12] were large cardiovascular outcome trials (CVOTs) with prespecified microvascular endpoints, whereas REWIND’s analysis [13] was post hoc, and Gullaksen’s study [14] was a small mechanistic trial with short follow-up (32 weeks). Retinopathy assessment also differed considerably: clinical complications in SUSTAIN-6 [11], patient-reported or investigator-reported events in EXSCEL [12], stratified analyses in REWIND [13], and physiologic markers such as retinal oxygenation in Gullaksen [14]. Follow-up duration ranged from less than a year to more than five years, influencing event capture. Importantly, none of the trials were primarily designed to test retinopathy progression, limiting statistical power and consistency across findings.

In this context, our risk-of-bias assessment provides additional nuance: although SUSTAIN-6 was a rigorously conducted trial with overall low risk of bias, its DR signal must be interpreted cautiously because retinopathy outcomes were captured only as adverse event reports rather than prespecified endpoints. By contrast, EXSCEL showed low risk across all domains, strengthening confidence in its neutral findings, while REWIND and Gullaksen’s analyses, though methodologically sound, were limited by their post hoc and mechanistic nature, respectively. Taken together, these methodological constraints highlight the challenge of drawing firm conclusions on DR risk, despite robust and reliable evidence for nephropathy benefit.

The renal benefits of GLP-1 receptor agonists likely reflect multifactorial mechanisms, including reductions in albuminuria, improvements in blood pressure and body weight, and possible direct effects on endothelial function and renal hemodynamics [15-17]. In contrast, the retinopathy worsening signal observed in SUSTAIN-6 [11] may be best explained by the phenomenon of early worsening - a transient increase in DR risk associated with rapid HbA1c reduction in patients with pre-existing disease, well documented in the Diabetes Control and Complications Trial (DCCT). Gullaksen et al. [14] provide additional insight, showing a small but significant increase in retinal thickness with semaglutide, though without corresponding changes in oxygenation or autoregulation, raising questions about structural versus functional retinal effects. These mechanistic considerations suggest that GLP-1 RAs may pose little intrinsic retinal toxicity, but that the pace of glycemic improvement and baseline DR status likely determine observed clinical outcomes [18].

Our findings align with broader evidence showing robust renal benefits of GLP-1 receptor agonists, echoing the well-established effects of sodium glucose cotransporter 2 (SGLT2) inhibitors, which consistently reduce albuminuria and slow kidney function decline [19]. Unlike GLP-1 RAs, however, SGLT2 inhibitors have shown no signal for retinopathy progression [20], reinforcing the uncertainty of the SUSTAIN-6 finding [11]. Observational studies and meta-analyses pooling GLP-1 RA trials largely suggest no overall increased risk of diabetic retinopathy, supporting the interpretation that the SUSTAIN-6 signal reflects context-specific rather than drug-class harm. This creates a clinical dilemma: in patients with advanced DR, clinicians must weigh the small but possible risk of worsening retinopathy against the clear renal and cardiovascular benefits that make GLP-1 RAs a valuable therapy [21].

The strengths of this review lie in its focus on randomized controlled trial evidence, which provides the highest-quality data available, and the application of structured risk of bias assessment to ensure critical appraisal. The inclusion of both large CVOTs and mechanistic studies allows for a balanced synthesis of clinical and physiologic evidence. However, key limitations include the reliance on only four eligible trials, most of which report secondary or post hoc analyses of microvascular endpoints not prespecified as primary outcomes. The small number of DR events and the heterogeneity of outcome definitions and follow-up durations further restrict the strength of conclusions, underscoring the need for more targeted research.

From a clinical standpoint, the evidence strongly supports GLP-1 RAs as renal-protective agents, making them attractive options for patients with type 2 diabetes at high risk of kidney disease [22]. Nevertheless, the uncertainty surrounding their impact on retinopathy means that caution is warranted in patients with pre-existing DR, particularly when initiating therapy with rapid HbA1c reductions [23]. In such cases, close ophthalmologic monitoring may mitigate potential risks while allowing patients to benefit from proven cardiovascular and renal protection. These findings emphasize the importance of a multidisciplinary approach, integrating endocrinology and ophthalmology care to optimize outcomes in complex patients [24].

Future research should focus on dedicated RCTs with retinopathy progression as a primary endpoint, which, to date, are lacking. Longitudinal mechanistic studies are needed to disentangle structural changes (e.g., retinal thickness) from functional alterations such as oxygenation and autoregulation, building on exploratory trials like Gullaksen et al. [14]. Comparative effectiveness studies directly evaluating GLP-1 RAs versus SGLT2 inhibitors in microvascular outcomes would further clarify therapeutic choices. Finally, real-world registry data could provide valuable insight into rare but clinically relevant retinopathy events, complementing trial evidence and guiding safe implementation of GLP-1 RAs in high-risk populations.

## Conclusions

The GLP-1 receptor agonists offer convincing renal protection and are an important therapeutic option for patients with type 2 diabetes, particularly those with high cardiovascular and renal risk. Their impact on diabetic retinopathy, however, remains inconclusive. The retinopathy signal seen in SUSTAIN-6 highlights the need for caution in patients with advanced DR but does not justify withholding these agents, provided patients are monitored appropriately. Overall, the balance of evidence favors their use, with careful clinical judgment ensuring that patients gain maximal cardiovascular and renal benefit without undue risk of ocular harm.
